# Supplementary material for: Integrating Oxygen and 3D Cell Culture System: A Simple Tool to Elucidate the Cell Fate Decision of hiPSCs
Source: Int J Mol Sci. 2022 Jun 30;23(13):7272. doi: 10.3390/ijms23137272 (PMC9266965; doi:10.3390/ijms23137272)
Supplement: Supplementary file 1 [file ijms-23-07272-s001.zip › ijms-1729580-supplementary.pdf]

Supplementary Material

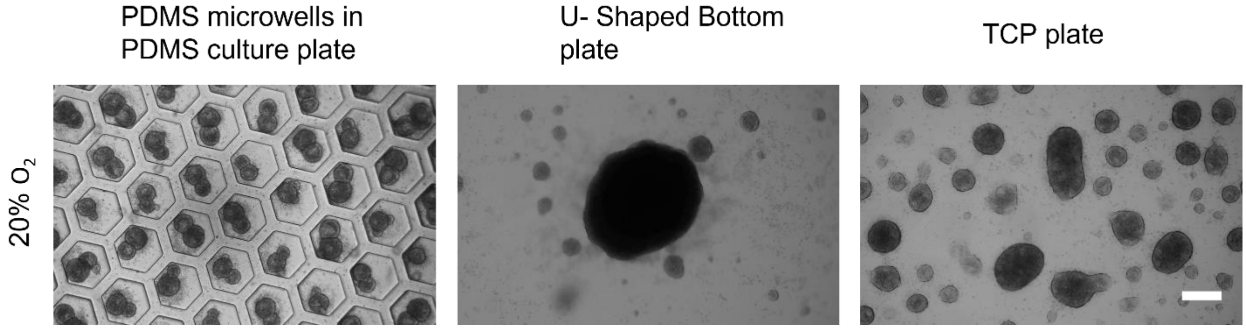

Figure S1. Morphology of the aggregates in PDMS microwells inserted in PDMS culture plate, 96 U- Shaped bottom plate and Tissue culture plastic (TCP) plate. Scale bar: 200 μm.
